# Deep learning model integrating positron emission tomography and clinical data for prognosis prediction in non-small cell lung cancer patients

**DOI:** 10.1186/s12859-023-05160-z

**Published:** 2023-02-06

**Authors:** Seungwon Oh, Sae-Ryung Kang, In-Jae Oh, Min-Soo Kim

**Affiliations:** 1 Department of Mathematics and Statistics, Chonnam National University, Gwangju, Republic of Korea; 2 Department of Nuclear Medicine, Chonnam National University Medical School and Hwasun Hospital, Hwasun, Jeonnam Republic of Korea; 3 Department of Internal Medicine, Chonnam National University Medical School and Hwasun Hospital, Hwasun, Jeonnam Republic of Korea

**Keywords:** Deep learning, Survival prediction, Lung cancer, Multimodal learning, FDG PET

## Abstract

**Background:**

Lung cancer is the leading cause of cancer-related deaths worldwide. The majority of lung cancers are non-small cell lung cancer (NSCLC), accounting for approximately 85% of all lung cancer types. The Cox proportional hazards model (CPH), which is the standard method for survival analysis, has several limitations. The purpose of our study was to improve survival prediction in patients with NSCLC by incorporating prognostic information from F-18 fluorodeoxyglucose positron emission tomography (FDG PET) images into a traditional survival prediction model using clinical data.

**Results:**

The multimodal deep learning model showed the best performance, with a C-index and mean absolute error of 0.756 and 399 days under a five-fold cross-validation, respectively, followed by ResNet3D for PET (0.749 and 405 days) and CPH for clinical data (0.747 and 583 days).

**Conclusion:**

The proposed deep learning-based integrative model combining the two modalities improved the survival prediction in patients with NSCLC.

## Background

Despite the recent development of novel treatment strategies, lung cancer is the leading cause of death worldwide. The 2-year and 5-year survival rates of lung cancer patients in the United States are low at approximately 30% and 20%, respectively [1]. The prediction of patient outcomes, such as the overall survival (OS), is important for guiding the treatment decision making. However, the current practice in predicting a prognosis is unsatisfactory. The prediction of OS following lung cancer diagnosis using tumor-node-metastasis (TNM) staging alone is the current practice in many hospitals [2]. The TNM stage has been used extensively by most physicians to roughly predict a patient outcome; however, heterogeneity within stage groups influences patient outcomes. Although various prognostic factors have been investigated for a more accurate survival prediction with advances in medical examinations, risk stratification of individual patients for precision medicine is still limited. One of the important reasons for this limitation is the difficulty in integrating different types of data containing prognostic information. This hurdle cannot be addressed using the traditional Cox proportional hazards model (CPH), a standard method for survival analysis in the medical field [3].

During the last few decades, a radiomic texture analysis with CPH has been actively investigated for survival prediction in patients [4]. The traditional radiomic texture analysis was based on extracting manually designed features (handcrafted features) from a manually or automatically segmented region of interest [5, 6]. However, there are limitations in extracting prognostic information from high-dimensional medical images using traditional radiomics models with handcrafted features [7–10]. Moreover, handcrafted feature extraction using traditional radiomics is laborious and time-consuming. Deep learning-based survival prediction models have recently outperformed traditional feature extraction methods, particularly when working with high-dimensional medical images [11–15].

Deep learning has also revolutionized image recognition. A convolution neural network (CNN), which is composed of multiple convolutional and pooling layers, is the dominant framework for image recognition [16]. A CNN builds layers of features while maintaining spatial information by receiving raw image input. The important aspect of a CNN is to see parts rather than the entire image and to make use of the association between one pixel of the image and the surrounding pixels. However, a deeper layer causes gradient vanishing and explosion problems, and a ResNet model using a shortcut method that adds residuals to the network has been developed. Therefore, the CNN model has expanded its applications to various tasks such as classification, detection, segmentation, and prognostic prediction in the medical image field [16–18].

F-18 fluorodeoxyglucose positron emission tomography (FDG PET) imaging, a type of functional whole-body imaging, is known to be a promising tool for prognostic prediction in patients with lung cancer. FDG PET provides information on disease pathophysiology that might be difficult to contain in clinical data [19]. We aimed to improve the prediction of the survival times in patients with non-small cell lung cancer (NSCLC), which accounts for the majority of all lung cancer, using a multimodal deep learning approach that integrates different types of medical data, including clinical variables and whole-body FDG PET images.

## Results

### Data preparation

Clinical variables and FDG PET images were collected from patients who were diagnosed with and treated for NSCLC between January 2011 and December 2017 at Chonnam National University Hwasun Hospital. Clinical data and PET images were obtained at almost the same time as the lung cancer diagnosis. FDG PET/computed tomography (CT) scans were obtained according to standardized imaging protocols at our institution using two types of PET/CT scanners. To test the generalization, PET images were derived from two types of PET/CT scanners: Discovery ST (GE Medical Systems, Milwaukee, WI, USA) and Discovery 600 (GE Medical Systems, Milwaukee, WI, USA). The three-dimensional (3D) PET images (whole-body axial images) had an image matrix of 128 × 128 × 427. Because coronal maximum intensity projection (MIP) images of FDG PET have shown promising results for survival prediction in patients with NSCLC [20], coronal MIP PET images were also obtained for comparison with 3D PET images. MIP PET images were obtained by projecting voxels with maximum intensity in parallel from the viewpoint to the coronal plane. Patients without any clinical factors or adequate pretreatment F-18 FDG PET/CT were excluded from the present study. Therefore, the datasets did not contain missing data. A treatment strategy for each patient, as determined by the multidisciplinary team, was recommended to the patients. This study was approved by the Institutional Review Board of our institution (CNUHH-2019-194).

A total of 2687 NSCLC patients (2005 men and 682 women, with a mean age of 67.95 ± 9.63 years) were included in this study. The datasets were split into two groups, 80% for training and 20% for testing. The patient characteristics for each dataset are listed in Table 1. There were no statistically significant differences among the features of each set based on a t-test for continuous variables and a Chi-square test for categorical variables. At the time of analysis, 1857 patients had died and 830 had been censored.

**Table 1:** Clinical features of training and test sets in the fold 1

Clinical features	Number of patients	Training set (n = 2149)	Test set (n = 538)	*p* value
Age	2687	67.97 ± 9.65	67.87 ± 9.57	0.837
*Sex*				
Male	2005 (74.6%)	1609 (80.2%)	396 (19.8%)	0.584
Female	682 (25.4%)	540 (79.2%)	142 (20.8%)	
*Histology*				
Adenocarcinoma	1501 (55.9%)	1197 (79.7%)	304 (20.3%)	0.986
Squamous cell carcinoma	1038 (1.5%)	834 (80.3%)	204 (19.7%)	
Large cell carcinoma	39 (1.5%)	31 (79.5%)	8 (20.5%)	
NOS	109 (4.1%)	87 (79.8%)	22 (20.2%)	
*Overall stage*				
I	595 (22.1%)	482 (81.0%)	113 (19.0%)	0.906
II	269 (10.0%)	213 (79.2%)	56 (20.8%)	
III	836 (31.1%)	667 (79.8%)	169 (20.2%)	
IV	987 (36.7%)	787 (79.7%)	200 (20.3%)	
*T stage*				
T1	575 (21.4%)	457 (79.5%)	118 (20.5%)	0.394
T2	1066 (39.7%)	852 (79.9%)	214 (20.1%)	
T3	544 (20.2%)	426 (78.3%)	118 (21.7%)	
T4	502 (18.7%)	414 (82.5%)	88 (175%)	
*N stage*				
N0	930 (34.6%)	753 (81.0%)	177 (19.0%)	0.096
N1	329 (12.2%)	273 (83.0%)	56 (17.0%)	
N2	709 (26.4%)	546 (77.0%)	163 (23.0%)	
N3	719 (26.8%)	577 (80.3%)	142 (19.7%)	
*M stage*				
M0	1700 (69.3%)	1363 (80.2%)	337 (19.8%)	0.773
M1	987 (36.7%)	786 (79.6%)	201 (20.4%)	
*Smoking history*				
Ever smoker	1861 (69.3%)	376 (20.2%)	1485 (79.8%)	0.763
Never smoker	826 (30.7%)	162 (19.6%)	664 (80.4%)	
Smoking amount	2687	29.44 ± 27.28	29.12 ± 27.11	0.809
*Dead status*				
Death	1857 (69.1%)	1485 (80.0%)	372 (20.0%)	0.974
Censored	830 (30.9%)	664 (80.0%)	166 (20.0%)	

The datasets were split into two groups, 80% for training and 20% for testing

*NOS* Non-specified; *T* Primary tumor; *N* Regional lymph node; *M* Distant metastasis

### Statistics and performance metrics

**Fig. 1:**
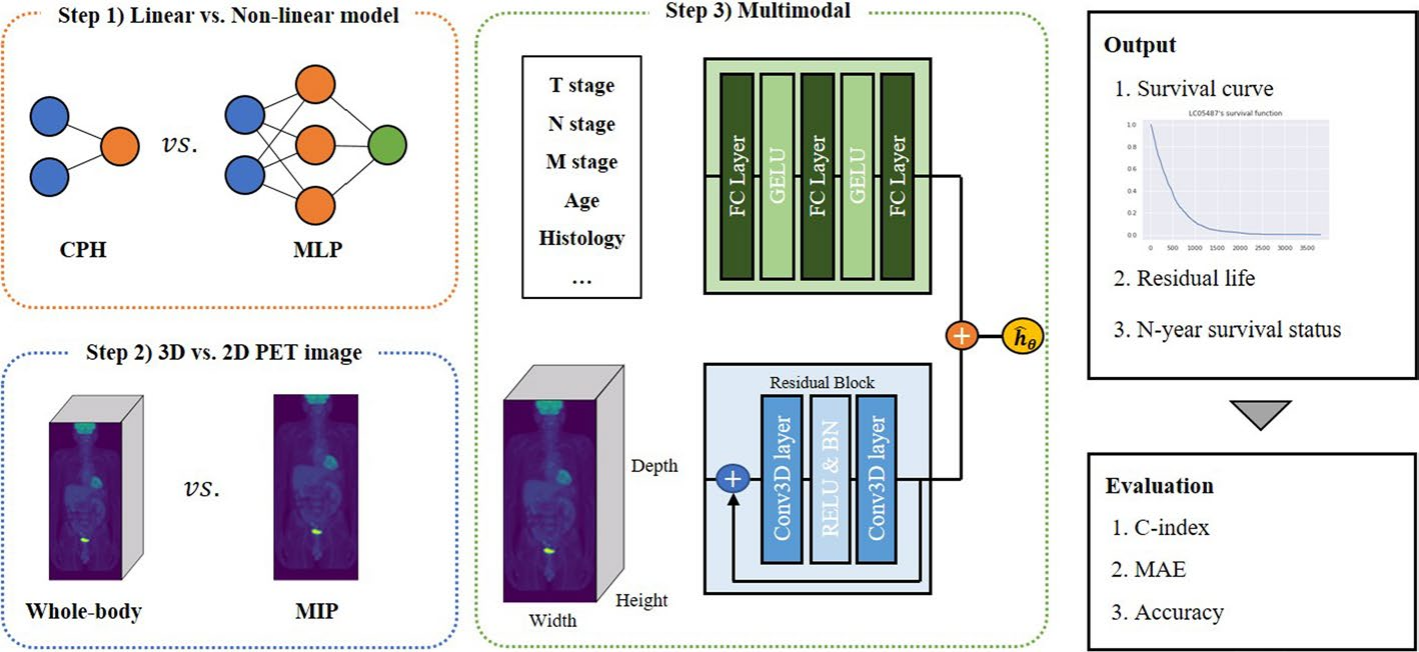
The structure of the proposed model and workflow. In step 1, the performances of the CPH and MLP model with DeepSurv were compared for use as a prediction model using clinical features. In step 2, the performance of a 3D CNN model with 3D PET images and a 2D CNN model with 2D MIP PET images were compared. In step 3, integration of the clinical features and image data for the proposed model occurs. The model performance was evaluated based on three metrics: C-index, MAE, and accuracy

The OS time was measured from the date of clinical diagnosis to the date of death. We predicted the absolute survival time and 2-year and 5-year survival status of the patients. We used the median residual life to predict the expected residual life expectancy (Fig. 1).

Baseline differences between the training and testing sets were assessed using a t-test for continuous variables and a chi-square test for categorical variables. Survival curves were generated using the Kaplan–Meier method and compared using the log-rank test [21]. Multivariate CPH regression analyses were conducted to estimate the prognostic effect of clinical features. Statistical significance was set at *p* < 0.05.

To compare the performance of the models in predicting the OS of an individual, we used C-index, MAE, and accuracy of the survival status. Owing to the presence of censoring in survival data, the frequently used evaluation metrics for regression, including the root mean squared error and *R*^*2*^, are inappropriate for estimating the prediction performance. Instead, specialized metrics such as the C-index and MAE are preferred for survival analysis [22]. The performance metrics were calculated and averaged using stratified five-fold cross-validation sets. The C-index is the fraction of all pairs of subjects whose predicted survival times are correctly ordered among all subjects that can be ordered. The C-index estimates the probability of the predicted survival time for each pair and evaluates whether each pair is of the same order as the actual survival time [23–26]. The C-index considers the relative risk of an event rather than the absolute survival times; therefore, we added the MAE to the performance metrics, which is the average of the differences between the predicted median residual lifetimes and actual observed OS times (ground truth) [22, 27]. Lower MAE values indicate a better model performance. We measured the MAE in the subgroup of uncensored patients (n = 1857) because the censored data underestimated the survival time [28]. The classification accuracy of 2- and 5-year survival status was also evaluated using the predicted residual life. A high accuracy indicates a better performance. Furthermore, we conducted a subgroup analysis to compare each model with the ground truth survival curve and MAE according to the overall stage.

### Experimental setup

All our experiments are conducted in a computer with an Intel(R) Xeon(R) Silver 4210R CPU and four Nvidia 3090 GPUs with 24 GB. The Adam optimizer was applied with a learning rate of 1e-4, a batch size of 6 per graphics processing units (GPU) according to the GPU memory capacity for 3D images, and a batch size of 125 for clinical data. Furthermore, the entire epoch was learned using callbacks with three digits of patience.

### Survival prediction models using clinical features

Table 2 presents the results of the multivariate CPH model. The model included nine clinical features, most of which are statistically significant risk factors for a poor OS. Older age, male sex, and advanced TNM stage were found to be independent predictors of a poor OS. Squamous cell carcinoma is associated with favorable survival outcomes.

**Table 2:** Multivariate Cox proportional hazard model for clinical variables associated with overall survival in non-small cell lung cancer patients

Clinical variable	Log hazard ratio	*p* value
Age	0.03	< 0.005
*Sex*		
Male	0.48	< 0.005
Female	0.00	
*Histology*		
Adenocarcinoma	− 0.19	0.12
Large cell carcinoma	− 0.05	0.72
Squamous cell carcinoma	− 0.70	0.04
NOS	0.00	
*T stage*		
T1	0.00	
T2	0.10	0.22
T3	0.21	0.02
T4	0.28	< 0.005
*N stage*		
N0	0.00	
N1	0.07	0.52
N2	0.20	0.05
N3	0.53	< 0.005
*M stage*		
M0	0.00	
M1	0.39	0.63
*Overall stage*		
I	0.00	
II	0.58	< 0.005
III	1.09	< 0.005
IV	1.36	0.1
*Smoking history*		
Ever smoker	0.00	
Non-smoker	− 0.06	0.13
Smoking amount	0.00	0.54

DeepSurv with an MLP model using clinical features consisted of 32, 64, and 128 nodes with two hidden layers, and a Gaussian error linear unit (GELU) was used as an activation function [29]. Unlike the rectified linear unit (RELU) function, which gives a difference according to the input of the gate, GELU is weighted according to the input value and is an active nonlinear function that is also used as an active function of MLP in the Vision Transformer (ViT) model [30]. A comparison of the DeepSurv MLP models with different nodes and the CPH model showed similar values for all models. However, the MLP with 64 nodes showed the best performance in terms of MAE and accuracy (Table 3). Therefore, we chose the DeepSurv MLP with 64 nodes for the final multimodal model.

**Table 3:** Performance comparison of survival prediction models using clinical features

Clinical model	MAE (days)	C-index	Classification accuracy of 2-year survival status	Classification accuracy of 5-year survival status
CPH	583 ± 37	**0.747 ± 0.01**	0.610 ± 0.05	0.868 ± 0.02
MLP {32 × 32}	506 ± 66	0.744 ± 0.01	0.731 ± 0.03	0.894 ± 0.03
MLP {64 × 64}	**463 ± 81**	0.745 ± 0.01	**0.740 ± 0.02**	**0.913 ± 0.03**
MLP {128 × 128}	500 ± 74	**0.747 ± 0.01**	0.730 ± 0.02	0.902 ± 0.03

The best score in each column is highlighted in bold

*MAE* Mean absolute error; *C-index* Harrell’s concordance index; *CPH* Cox proportional hazards; *MLP* multilayer perceptron

### Survival prediction models using PET images

For survival prediction using 2D MIP images, among ResNet with 18, 34, and 50 layers, the performance improved further as the number of layers increased. ResNet with 50 layers (ResNet-50) showed a better performance in terms of the MAE and classification accuracy than CPH, but not the C-index. For survival prediction using 3D PET images, 3D CNN ResNet3D models with 10, 18, and 34 layers were compared. Because whole-body PET images have a large volume, ResNet variants with a relatively low network depth (layers) were evaluated. The CNN models using 3D PET images showed better performance in all metrics than models using 2D PET images. ResNet3D with 34 layers (ResNet3D-34) achieved the best performance among all PET models (Table 4). Therefore, we chose the ResNet3D-34 using 3D PET images for the final multimodal model.

**Table 4:** Performance comparison of the survival prediction of convolutional neural network (CNN) models using positron emission tomography (PET) images

PET model	MAE (days)	C-index	Classification accuracy of 2-year survival status	Classification accuracy of 5-year survival status
*ResNet (MIP)*				
18 layers	447 ± 26	0.710 ± 0.02	0.719 ± 0.03	0.920 ± 0.01
34 layers	470 ± 14	0.713 ± 0.02	0.699 ± 0.03	0.908 ± 0.01
50 layers	423 ± 22	0.717 ± 0.01	0.724 ± 0.03	0.924 ± 0.01
*ResNet3D*				
10 layers	440 ± 33	0.729 ± 0.01	0.726 ± 0.02	0.917 ± 0.01
18 layers	429 ± 15	0.740 ± 0.01	0.733 ± 0.02	0.915 ± 0.01
34 layers	**405 ± 29**	**0.749 ± 0.02**	**0.751 ± 0.02**	**0.928 ± 0.01**

The best score in each column is highlighted in bold

*MAE* Mean absolute error; *C-index* Harrell’s concordance index; *MIP* Maximum intensity projection

### Multimodal deep learning

The DeepSurv MLP model using clinical features showed better performance than CPH model in terms of the MAE and classification accuracy of 2- and 5-year survival status. ResNet3D-34 using PET images showed a similar performance as the CPH model in terms of the C-index but a much better performance than the CPH in terms of the MAE and classification accuracy of 2- and 5-year survival status. Therefore, we proposed multimodal model combining ResNet3D-34 and MLP with 64 nodes and two layers. The proposed multimodal model showed the best performance in all prediction models. The C-index was the highest in the multimodal model, reaching 0.756 ± 0.01 under a five-fold cross validation. In addition, the MAE also showed the smallest error (approximately 1 year). Furthermore, the 2- and 5-year classification accuracies were the highest, reaching 0.743 ± 0.02 and 0.933 ± 0.01, respectively, with the proposed model (Table 5).

**Table 5:** Performance comparison of models using clinical data, positron emission tomography (PET) data, or dual modality

Data	Model	MAE (days)	C-index	Classification accuracy of 2-year survival status	Classification accuracy of 5-year survival status
Clinical data	CPH	583 ± 37	0.747 ± 0.01	0.610 ± 0.05	0.868 ± 0.02
	MLP {64 × 64}	463 ± 81	0.745 ± 0.01	0.740 ± 0.02	0.913 ± 0.03
PET (MIP images)	ResNet-50	423 ± 22	0.717 ± 0.01	0.724 ± 0.03	0.924 ± 0.01
PET (whole-body axial images)	ResNet3D-34	405 ± 29	0.749 ± 0.02	**0.751 ± 0.02**	0.928 ± 0.01
Clinical data + PET (whole-body axial images)	Multimodal	**399 ± 27**	**0.756 ± 0.01**	0.743 ± 0.02	**0.933 ± 0.01**

The best score in each column is highlighted in bold

*MAE* Mean absolute error; *C-index* Harrell’s concordance index; *CPH* Cox proportional hazards; *MLP* Multilayer perceptron; *MIP* Maximum intensity projection

**Fig. 2:**
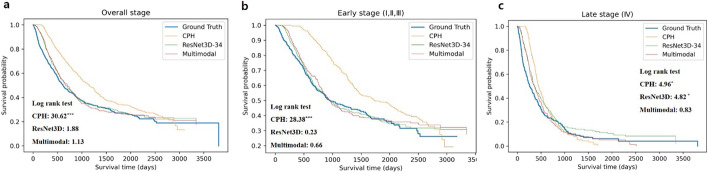
Survival curves of ground truth and each model in the test set. **a** Survival curves for each model at all stages. **b** Survival curves of each model in the early stages (I, II, and III). **c** Survival curves of each model in the advanced stage (IV). *p < 0.05, ***p < 0.001

Figure 2 shows the Kaplan–Meier curves comparing the distribution of the ground truth of the actual survival time and the predicted survival times using each model in the test set. Log-rank tests were conducted to evaluate the similarity of the survival distributions. There were no statistically significant differences between the ground truth and ResNet3D model (*p* = 0.17) or between the ground truth and multimodal model (*p* = 0.29). However, there was a significant difference between the ground truth and CPH (*p* < 0.001). In the early stage (I, II, III) of NSCLC patients, the CPH model (*p* < 0.001) showed a statistically significant difference from the actual survival curve, whereas ResNet3D (*p* = 0.629) and the proposed multimodal model (*p* = 0.416) showed no statistically significant difference. However, in the advanced stage (IV), the CPH (*p* = 0.026) and ResNet3D (*p* = 0.028) models showed a statistically significant difference from the actual survival curve, whereas the proposed multimodal model (*p* = 0.362) did not. Prediction models that use PET images as a portion of the input data provided more accurate survival predictions than the prediction model using only clinical data in early-stage NSCLC patients. In addition, the proposed multimodal model showed no significant difference from the actual survival curve and provided a more accurate survival prediction than other models in all stages of NSCLC patients.

**Fig. 3:**
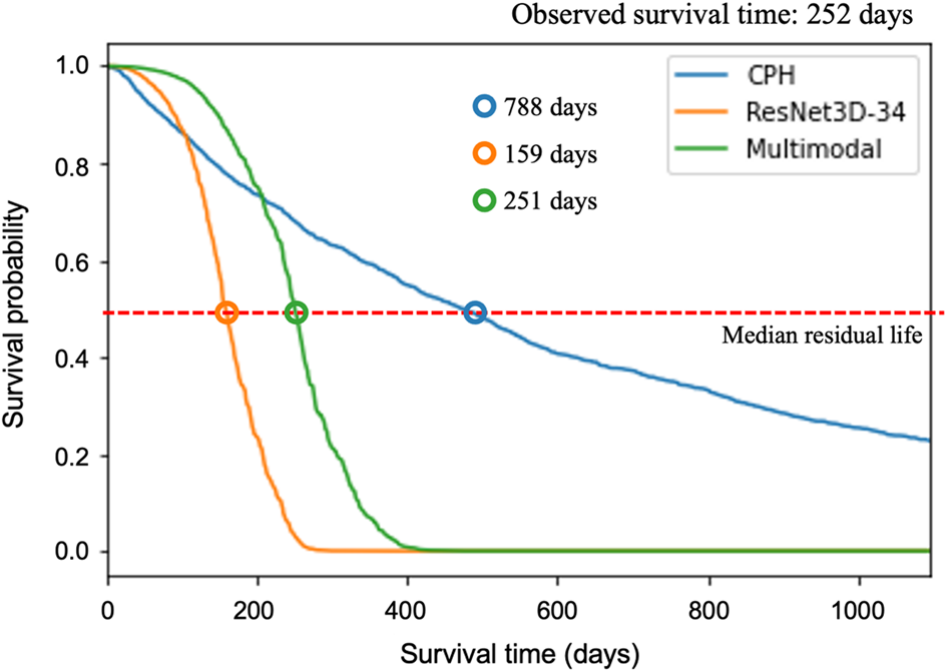
Prediction of survival curves of each model in a representative patient

The survival curves for each patient with NSCLC were estimated from the predicted hazard ratios. Figure 3 shows the results of estimating individual survival curves in a representative 60-year-old male patient with stage III NSCLC without a history of smoking. The patient’s actually observed survival time was 252 days. The predicted survival time of each model was estimated using the median residual life. The residual lifetimes predicted by the CPH, ResNet3D, and multimodal models were 788, 159, and 251 days, respectively. The most accurate model used to predict the actual survival time was multimodal model, which showed the smallest error (1 day) in comparison with ResNet3D (93 days) and CPH (536 days).

**Fig. 4:**
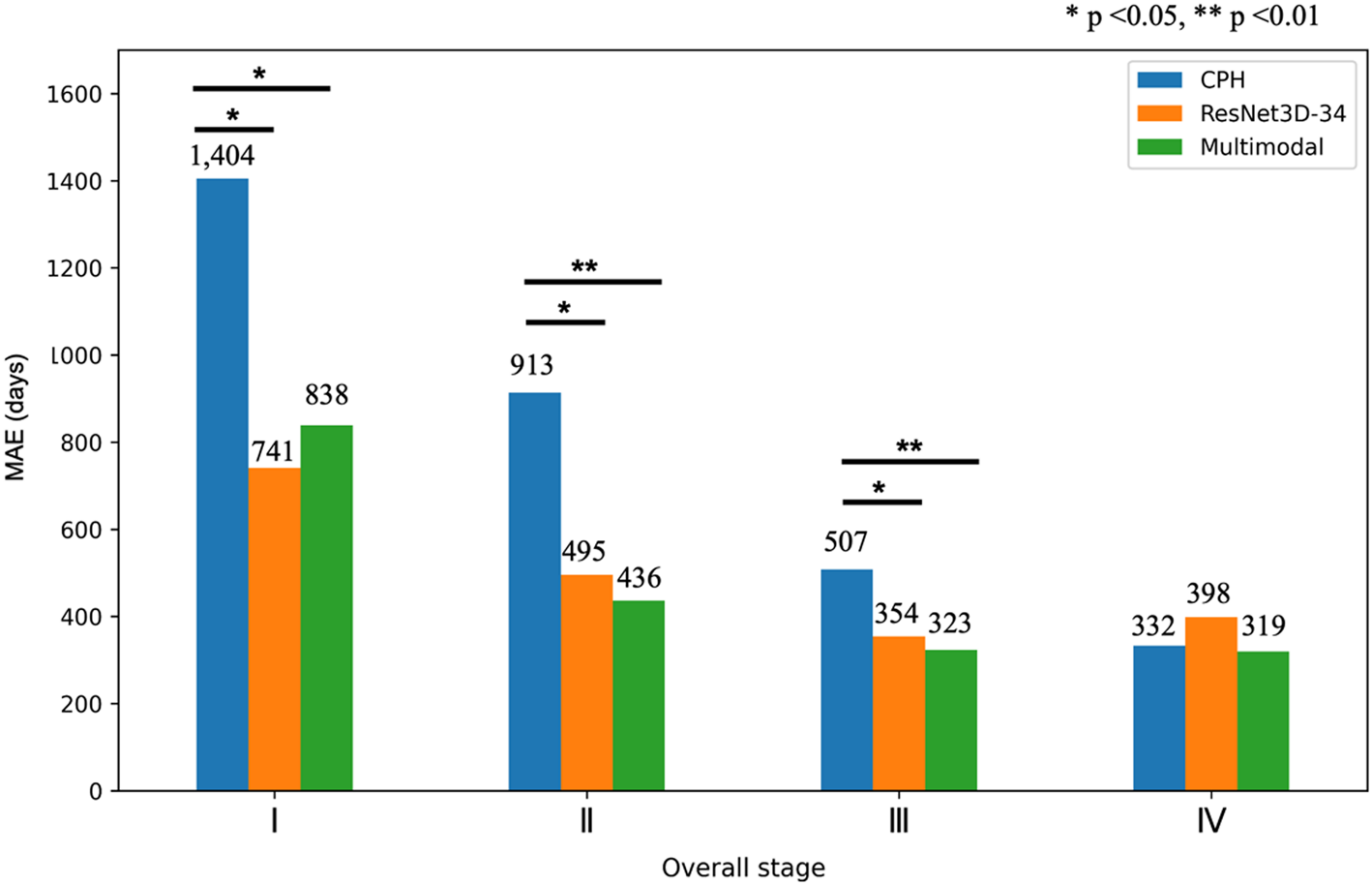
Comparison of mean absolute error (MAE) in each stage. *p < 0.05, **p < 0.01

In the subgroup analysis of the MAE in patients according to overall stage, the advantages of the model using PET data (ResNet3D-34 and multimodal model) were more prominent than those of the model using clinical data (CPH) in the early stage (I, II, and III) (Fig. 4). In the early stage, the ResNet-34 and multimodal model showed a statistically significant difference from CPH. The MAE of the CPH showed a larger error in the early stage than in the advanced stage. Additional prognostic information from PET images might be advantageous, particularly in early-stage NSCLC patients.

## Discussions

The prediction of a prognosis in individual patients is important for predicting the effectiveness of a treatment and improving patient care [31]. In the present study, a multimodal deep learning model is proposed that integrates two heterogeneous modalities (clinical data and 3D PET images) with joint fusion to predict the OS time in NSCLC patients. The integrative multimodal model showed an improved prognostic performance compared to the traditional CPH model using clinical data, a ResNet model using 2D PET images, and a ResNet3D model using 3D PET images. The proposed model seems to effectively combine the information inherent in the two different modalities and reflects them in the survival prediction. This is probably because, unlike ResNet2D, ResNet3D allows learning additional information, such as the spatial context around the tumors. Furthermore, ResNet3D handles a relatively small axial area close to the tumor such that the level of attention is not distracted by uninformative non-tumor areas in the images [32, 33]. As the ResNet3D model outperformed other 3D-CNN models comparing C3D and RGB-I3D models in Kinetics, a large-scale video dataset [33], our results were consistent with the previous study.

Traditional radiomic approaches for predicting cancer prognosis using imaging data have been actively investigated [4, 34]. However, the handcrafted feature extraction of radiomics is laborious and time-consuming and cannot use the complete information of the images. Because deep learning-based models have shown a good performance in terms of image classification, localization, detection, segmentation, and registration, deep learning-based survival prediction has been investigated to overcome these limitations; however, this approach has not been fully investigated [35]. Whereas traditional CPH predicts the hazard function and requires specific assumptions to evaluate the survival time, the proposed model directly predicts the individual survival time (residual life). Direct survival time prediction, rather than a hazard function or distribution function, provides a more intuitive interpretation of the prognostic predictions [36].

In the present study, both 2D and 3D PET images were evaluated as input data for survival prediction. The prediction model of 3D PET images showed a much better performance than that of 2D MIP images [37, 38]. MIP is a common visualization method that can be used to visualize 3D images by converting them into 2D images [39]. MIP PET images project voxels with maximum intensity in a parallel manner from the viewpoint to the plane. Although MIP images allow a reduction of the data size and computing power during training, they might be limited to reflecting the spatial information of the tumor, which contains useful prognostic information. The use of whole 3D medical images might be more robust than 2D images for prognostic prediction in cancer patients [17].

The present study has certain limitations. First, the number of features in the clinical data was limited because it was difficult to collect medical data through electronic medical records. However, we included essential clinical risk factors that were preferentially collected and readily used as prognostic factors in the real world. The TNM stage alone is often considered as a prognostic factor when making decisions regarding treatment and management owing to the lack of an appropriate model incorporating information from different modalities. Moreover, we included major risk factors for NSCLC, such as age, sex, histology, smoking history, and the TNM stage. Second, PET images without lesion annotation were used. Although a lesion annotation might have improved the predictive performance of deep learning models, lung cancer patients may have multiple metastatic lesions, ranging from several to hundreds. It takes a significant amount of time and effort by physicians to annotate such lesions. Instead, we attempted to improve the accuracy and generalize the model by collecting data from a relatively large number of patients. Finally, the present study still has room for performance improvements by using state-of-art CNN models. Variants of ResNet such as 3D densely connected convolutional network (3D-DensNet) and ResNet(2 + 1)D have been proposed and outperformed ResNet3D in imaging analysis [40–42]. Further research is necessary to address the challenges predicting prognosis using state-of-art CNN models for medical imaging applications.

## Conclusion

The results of the present study indicate that deep learning model integrating clinical data and PET image data should improve prognostic prediction power in NSCLC patients, especially in patients with early stage. The proposed multimodal deep learning model can successfully integrate different types of medical data and provide intuitive prognostic prediction results to physicians and NSCLC patients.

## Method

The modeling process that combines the two modalities is shown in Fig. 1. First, we compared the performance of DeepSurv with that of traditional CPH to choose a suitable model for clinical data. DeepSurv is a multilayer perceptron (MLP) adapted for survival analysis, which is a form of a feedforward deep neural network (DNN). DeepSurv predicts the effects of clinical covariates on the hazard rate parameterized by the weight of the network. The loss function for DeepSurv includes a negative log partial likelihood from the CPH and regularization term. The open-source code DeepSurv by Katzman et al. was used [43]. To optimize the hyperparameter of DeepSurv, three layers (32, 64, and 128 nodes) of MLP were compared using the Harrell’s concordance index (C-index) and mean absolute error (MAE).

Then, we compared the predictive ability of ResNet3D for 3D PET images with ResNet for two-dimensional (2D) MIP images. Because ResNet contains shortcut connections that turn the network into its counterpart residual version and allows stacked layers to fit the residual mapping, we proposed ResNet to extract features of PET images [17]. For survival prediction using 2D MIP images, ResNet models with 18, 34, and 50 layers were compared. For survival prediction using 3D PET images, 3D CNN ResNet3D models with 10, 18, and 34 layers were compared. We used a model structure that uses batch normalization and a RELU as an activation function after each convolution layer. The size of the convolution kernel is (3 × 3 × 3), two stride convolution layers were used for downsampling, and adaptive average pooling was applied to make the last fully connected layer [33]. Final multimodal model was constructed by combining CNN of optimal parameters in PET and DNN of optimal parameters in clinical data [44].

## Data Availability

The data sets used in this study can be downloaded from https://aihub.or.kr/aihubdata/data/view.do?currMenu=115&topMenu=100&aihubDataSe=realm&dataSetSn=228.

## Abbreviations

NSCLC: Non-small cell lung cancer
CPH: Cox proportional hazards
FDG PET: F-18 fluorodeoxyglucose positron emission tomography
CT: Computed tomography
OS: Overall survival
TNM: Tumor-node-metastasis
MIP: Maximum intensity projection
RELU: Rectified linear unit
GELU: Gaussian error linear unit
ViT: Vision transformer
MLP: Multilayer perceptron
DNN: Deep neural network
C-index: Harrell’s concordance index
MAE: Mean absolute error
