# Correction: Deep learning model integrating positron emission tomography and clinical data for prognosis prediction in non-small cell lung cancer patients

**DOI:** 10.1186/s12859-023-05359-0

**Published:** 2023-06-01

**Authors:** Seungwon Oh, Sae-Ryung Kang, In-Jae Oh, Min-Soo Kim

**Affiliations:** 1 Department of Mathematics and Statistics, Chonnam National University, Gwangju, Republic of Korea; 2 Department of Nuclear Medicine, Chonnam National University Medical School and Hwasun Hospital, Hwasun, Jeonnam Republic of Korea; 3 Department of Internal Medicine, Chonnam National University Medical School and Hwasun Hospital, Hwasun, Jeonnam Republic of Korea

**Correction: BMC Bioinformatics (2023) 24:39** https://doi.org/10.1186/s12859-023-05160-z

Following publication of the original article [1], it was reported that the article entitled “Deep learning model integrating positron emission tomography and clinical data for prognosis prediction in non-small cell lung cancer patients” was published in the regular issue of this journal instead of in the supplement issue.

The details of the supplement in which this article ought to have been published are given below:

**About this supplement**

This article has been published as part of BMC Bioinformatics Volume 23 Supplement 9, 2022: Proceedings of the 15th International Conference on Data and Text Mining in Biomedical Informatics (DTMBIO 2021). The full contents of the supplement are available online at https://bmcbioinformatics.biomedcentral.com/articles/supplements/volume23-supplement-9.

The publisher apologizes for any inconvenience caused.
